# Ketogenic diet, adenosine, and dopamine in addiction and psychiatry

**DOI:** 10.3389/fnut.2025.1492306

**Published:** 2025-03-10

**Authors:** David N. Ruskin, Luis A. Martinez, Susan A. Masino

**Affiliations:** Trinity College, Hartford, CT, United States

**Keywords:** ketogenic diet, adenosine, dopamine, psychiatric disorder, addiction, mental health, metabolic psychiatry

## Abstract

Adhering to the ketogenic diet can reduce or stop seizures, even when other treatments fail, via mechanism(s) distinct from other available therapies. These results have led to interest in the diet for treating conditions such as Alzheimer’s disease, depression and schizophrenia. Evidence points to the neuromodulator adenosine as a key mechanism underlying therapeutic benefits of a ketogenic diet. Adenosine represents a unique and direct link among cell energy, neuronal activity, and gene expression, and adenosine receptors form functional heteromers with dopamine receptors. The importance of the dopaminergic system is established in addiction, as are the challenges of modulating the dopamine system directly. A mediator that could antagonize dopamine’s effects would be useful, and adenosine is such a mediator due to its function and location. Studies report that the ketogenic diet improves cognition, sociability, and perseverative behaviors, and might improve depression. Many of the translational opportunities based on the ketogenic diet/adenosine link have come to the fore, including addiction, autism spectrum disorder, painful conditions, and a range of hyperdopaminergic disorders.

## Introduction

Metabolic therapy with the ketogenic diet (KD) has been used successfully to treat epilepsy in adults and children for over 100 years (1). Adhering to this high-fat, low-carbohydrate protocol can reduce or even stop seizures – even when all other treatments fail, and some pediatric patients are able to discontinue the KD and remain seizure-free (2–6). This effect is also found with laboratory animals (7), indicating a disease-modifying, antiepileptogenic effect found only weakly in some but not present at all in most anticonvulsant medications (8, 9). These observations indicate that this metabolic therapy works via mechanism(s) distinct from other available therapies—and demonstrate clinically that a KD may permanently restore normal brain function.

The proven, long-term efficacy in epilepsy has led to interest in the KD’s mechanisms for preventing and treating multiple conditions (such as diabetes), but particularly in other neurological conditions such as Parkinson’s disease, Alzheimer’s disease, and multiple sclerosis (10), as well as conditions where seizures are often comorbid. Several types of behavioral evidence predict benefits of a KD in reducing common comorbidities other than seizures such as depression (11, 12) and anxiety (11, 13). Most studies report that the KD improves cognition (13–19), improves sociability and repetitive behaviors (20–23), and reduces nociception (24–26): all behavioral endpoints with relevance to dopamine-related behaviors (see below), including perseverative behaviors and potentially chronic pain – thought to share multiple mechanisms and comorbidities with addiction (27). Importantly, KD-related behavioral improvement in children with epilepsy is not solely due to seizure reduction (13, 28–32), thus indicating therapeutic benefits that are uncorrelated with the primary anticonvulsant/antiepileptogenic effects.

Diverse lines of evidence point to the neuromodulator adenosine as a key mechanism underlying short and long-term therapeutic benefits of metabolic therapy with a KD. Adenosine is present throughout the extracellular space, and its levels increase with increased neural activity (33, 34) and a variety of physiological conditions (35). We put forth this hypothesis and its translational predictions in 2008 (36). Since then, we developed metabolic models and provided *in vitro* and *in vivo* evidence that KD feeding elevates brain adenosine (7, 36–39). More evidence has since accumulated (40–42), and many of the translational opportunities based on the KD/adenosine hypothesis have come to the fore, including pain, autism spectrum disorder, neuroprotection, and a range of hyperdopaminergic disorders (35, 36). Adenosine represents a unique and direct link among cell energy, neuronal activity, and gene expression and a direct functional relationship with dopamine. Here, we review several molecular/physiological actions of the KD by which the KD might influence addiction and psychiatric disorders, then delve into specific disorders with respect to KD treatment.

### Adenosine/dopamine interactions

The behavioral importance of the dopaminergic system is well-established – as are the challenges and limitations (side effects, limited therapeutic windows) of modulating the dopamine system directly. Dopamine release is clearly related to the reinforcing effect of drugs of abuse, such as cocaine, which blocks re-uptake of dopamine and so increases extracellular levels of this neurotransmitter. The discussion below is largely focused on cocaine. Chronic use of this drug in people leads to a number of behavioral sequelae, including highly-motivated use even in the face of adverse consequences. Laboratory rodents chronically self-administering cocaine show similar behaviors (43), including no diminution of self-administration even in the face of a signal of impending footshock (43, 44). Remarkably, cocaine cravings increase over 60 days of withdrawal in rodents (45), in accordance with reports in human addiction. PET studies in cocaine-addicted patients show reduced D2 dopamine receptor levels in the basal ganglia and reduced metabolism in the cingulate gyrus and orbitofrontal cerebral cortex (46). Brain effects of cocaine progress with extended self-administration, with extension of metabolic changes from the limbic basal ganglia to the entire basal ganglia in Rhesus monkeys (47), and progressively elevated levels of brain-derived neurotrophic factor in the limbic basal ganglia and amygdala; this protein causes long-lasting amplification of cocaine seeking (48).

A mediator that could interfere with the effects of dopamine (without blocking it completely) would be extremely useful, and adenosine is such a mediator due to its function and its location. Manipulating the adenosine system is common – caffeine, a non-selective antagonist for adenosine A_1_ receptors (A_1_R) and adenosine A_2_ receptors (A_2_R), is the most widely used psychoactive drug worldwide – and other adenosine antagonists are under consideration for neurodegenerative and psychiatric disorders (49, 50). Notably, subpopulations of richly dopamine-innervated basal ganglia neurons express either a combination of A_1_R and D1 dopamine receptors (D_1_R) or A_2A_R and D2 dopamine receptors (D_2_R) (51, 52), and these colocalized receptors form functional heteromers with antagonistic effects on 2nd messenger systems (53–58).

These oppositional relationships also appear at the behavioral level in rodents, in work often involving cocaine. For example, A_2a_R agonists decreased, whereas A_2a_R antagonists increased, acute cocaine-induced locomotion, in apparent opposition to the D_2_R (57). Selectively knocking out A_2a_R expression in striatal neurons enhances the locomotor response to cocaine or phencyclidine (58). Chronic caffeine in adolescence increases the locomotion to a challenge dose of cocaine or a D_2_R agonist in adulthood (59). Outside the brain, A_1_R and D_1_R oppositely influence spinal motor circuit output (60). Caffeine reduced the locomotor sensitization response to cocaine in a binge protocol (61). Given during a sensitization regimen, A_2a_R agonists decreased, whereas A_2a_R antagonists increased, the sensitized response to a later cocaine challenge (57). Alternatively, A_1_R or A_2a_R agonists given during the cocaine challenge but not during sensitization reduced the expression of cocaine sensitization, in a paradigm in which the adenosinergic drugs were directly infused into the basal ganglia (62).

In the conditioned place preference test, adolescent chronic caffeine enhanced the rewarding effect of cocaine in adulthood (59). An A_2a_R agonist reduced the reinforcing and motivational aspects of cocaine self-administration (63). A_1_R agonists inhibited cocaine- or D_1_R agonist-induced reinstatement of extinguished cocaine self-administration (64). Caffeine potentiated the seizure-inducing properties and lethality of cocaine and D-amphetamine (65). Also relevant to drug abuse, adenosine and dopamine (mostly the A_2a_R and D_2_R) differentially control motivation (66). Overall, there is an abundance of evidence that adenosine and dopamine receptors are in opposition in their influence on several types of behavior and cognition.

Some evidence suggests the KD alters dopamine directly. The dopamine metabolite homovanillic acid was reduced during KD feeding in pediatric epileptic patients in a study that used CSF as a proxy for tissue dopaminergic activity, though this effect did not differ with presence or absence of anticonvulsant response (67). In rats, tissue homovanillic acid (combined with another dopamine metabolite, dihydroxyphenylacetic acid) was elevated by the KD in cerebral cortex but not basal ganglia or midbrain (68). These differences could be explained by a number of factors, such as species differences, differences in subject maturity, differences in KD strength/composition, or the effective whole-brain sampling of CSF collection. A KD-based mechanism to moderate adenosine and/or dopamine systems would have obvious relevance to neurological conditions, including drug abuse (69, 70).

### Cerebrocortical hypometabolism versus energy replenishment

Hypotheses and clinical and basic research on the link between brain energy and mental health has been a rapidly developing field with case reports, reviews, protocols, and cutting-edge conferences helping to foster a robust and thriving community with real collaboration between patients and professionals (71–76). Compensating for the energy impairment due to ongoing hypometabolism may be a useful treatment for many diverse neurological conditions (77–80). Energy homeostasis – particularly changes in ATP and adenosine – is known to be relevant but poorly understood in neuroprotection, psychiatric disorders and addiction (81, 82). KDs supply a substrate (ketone bodies) for the citric acid cycle that elevates ATP and promotes mitochondrial function, including in impaired states (83–97).

Brain hypometabolism has been reported with alcohol and online gaming addiction (98), with stimulant abuse (99, 100), in Alzheimer’s disease and mild cognitive impairment (101, 102), and indeed even with normal aging (103). As a dynamic and energy-demanding organ, and as a survival mechanism, it makes sense that metabolism is reflected in neurological function and behavior and that mitigating metabolic dysfunction is a potent therapeutic strategy.

### Reduced hyperglycemia and/or inflammation

KDs produce a moderately low but very stable blood glucose (104–107), explaining why it is an effective treatment for diabetes (106, 108, 109). This stabilization of blood glucose may blunt the impact of well-known physiological effects of stress and/or dopamine-induced hyperglycemia (110), and therefore may help stabilize a range of mental states that are influenced by metabolic variability, particularly those that are triggered by or associated with hyperglycemia. Hyperglycemia causes inflammation (111, 112) and is associated with psychiatric re-hospitalization. Inflammation is a biomarker for and perhaps a cause of depression (113), and much evidence shows that KDs reduce inflammation in patients (114–117) and in pre-clinical models (118–121). Importantly, some animal studies found reduced inflammatory markers specifically in brain (122–125). KD feeding seems to limit neuroinflammation via several mechanisms (126).

## Disorders

### Addiction

Based on the relationships among adenosine, dopamine, and the KD, we recently investigated the possible moderating effects of KD treatment on the effects of repeated cocaine treatments (127). Five-week old male and female rats were placed on a KD or remained on normal rodent chow for 3 weeks. A well-established cocaine-sensitizing regimen was then applied: animals received once-daily injections of either saline or cocaine for seven consecutive days, followed by seven drug-free days, and then finally a challenge injection of cocaine. Assessments occurred in an automated system for measuring ambulatory (e.g., walking) and stereotyped (e.g., rearing) locomotor responses. KD feeding continued through the sensitization protocol. All animals receiving the daily cocaine injections showed the expected enhancement of the rearing response, but animals on the KD had a significantly mitigated enhancement. Unexpectedly, ambulatory activity did not sensitize at all in KD-fed animals. These effects of KD on locomotor activity were found in both sexes, and were only observed following injections of cocaine (not saline). A similar pattern was found with the challenge injection: KD treatment moderated the stereotypic response to the challenge. Interestingly, here sex was a factor, with this effect occurring in males only. Thus, KD feeding reduces both the responses to acute cocaine (day one of the sensitizing regimen; challenge day for saline-treated animals) and repeated cocaine. Considered together, these data were the first to show that KD treatment can modify behavioral responses to a monoaminergic stimulant, and suggest that KDs are a potential novel therapy for the treatment of addiction to these drugs. Based on prior studies, we posit that the effects of the KD in this paradigm could be mediated by an effect of adenosine on dopaminergic systems, likely in the basal ganglia.

More recently, the effects of KD treatment were tested in a conditioned place preference protocol, wherein animals learn to prefer a section of the experimental apparatus paired with, in this case, cocaine injections (128). KD feeding did not appear to modulate the acquisition of the cocaine-related place preference. However, when cocaine was withheld (i.e. extinction), mice on the KD more quickly lost the place preference. In addition, a cocaine priming injection after extinction reinstated the place preference only for the standard diet mice; mice that were on KD did not experience reinstatement. The authors hypothesized that the KD effects were via an adenosine/dopamine interaction, and suggested that KD treatment might be especially useful in preventing relapse.

Regarding the commonly abused drug ethanol, in rat models of dependence KD-fed animals made fewer lever presses to receive alcohol during acute withdrawal (129) and had reduced withdrawal symptoms (130, 131). In mice, both KD and a ketone monoester (which is metabolized to ketone bodies) reduced withdrawal symptoms even when treatment was started during withdrawal (132). Clinically, benzodiazepines are given to reduce withdrawal symptoms during detoxification: notably, patients eating a KD during treatment required significantly fewer or lower doses of benzodiazepines (129). Alcohol-related stimuli induced fewer or lower doses of “wanting” and more dorsal anterior cingulate gyrus activation in patients on a KD; neuroinflammatory markers were also reduced (129). An alcohol-dependent metabolism has switched from depending on glucose to depending on acetate; ketone bodies might normalize metabolism by replacing acetate (133). It had been hypothesized that alcohol addiction might relate to adenosine dysfunction in the basal ganglia (134) and a recent study provides direct evidence (135).

Adenosine is clearly involved in the effects of opiates. During a dependence-inducing regimen of morphine and during withdrawal, brainstem adenosine was reduced two-fold (136). During withdrawal, symptoms were reduced with an A_1_R agonist or an A_2a_R antagonist (136) or genetic inactivation of A_2a_R (137). Consistent with these results, KD feeding reduced symptoms of withdrawal from opiates in mice (138, 139). In addition, KD feeding reduced opiate self-administration (139) and hyperalgesia due to chronic opiate treatment (140). These results suggest a KD, through adenosine, might have some utility in opiate abuse. Conversely, a KD elevated locomotor responses and analgesia to oxycodone. This latter effect, however, could be partially explained by the antinociception due to the KD itself (25, 120).

Food cravings, binge eating being an extreme form, are often considered to be a naturalistic analog of drug abuse. Excessive glucose and insulin spikes are thought to modify the brain leading to addiction-like binge eating; KD feeding will temper such spikes (141). Two pilot studies of KD treatment to patients with food addiction/binge eating disorder underwent KD treatment, leading to significant reductions (142) or complete alleviation (143) of the disorder’s symptoms.

### Psychiatric disorders

A recent study found that KD treatment in 28 patients with severe refractory mental illness significantly improved psychotic symptoms and depression; virtually every patient improved on multiple scales (144). Twelve of the patients achieved clinical remission on the Clinical Global Impressions Scale. A majority of patients reduced number or dose of psychotropic medications (in a number of cases, diabetes-related medications were reduced or discontinued) (144). After discharge, 18 patients chose to remain or partially remain on the diet to maintain the psychiatric benefits. Subsequent studies have also found broad KD effectiveness in mental illness such as bipolar disorder and schizophrenia (145–151). Much evidence shows that KDs reduce inflammation in patients (114–117, 152) and in pre-clinical models (118–121). Reductions in inflammation might be particularly germane to depression (113).

An involvement of adenosine (specifically, an alteration in normal adenosine/dopamine antagonism) has long been postulated for schizophrenia (153–155), and adenosine modulators have been tried with some success in patients (156). More recent papers have highlighted abnormalities in adenosine receptor expression specifically in frontal cerebral cortex but not other adenosine receptor expressing regions (157, 158). In parallel, hypometabolism, limited to the frontal cerebral cortex, was indicated in schizophrenia by meta-analysis (159). Therefore, the KD might have beneficial effects via multiple mechanisms. One group found positive effects in an animal model of schizophrenia-like behavior (160–162). A very early attempt to use the KD in schizophrenic patients showed promise but was poorly controlled (163). More recently, beneficial results have been reported, but these are either case reports (164, 165) or have a low number of subjects (five schizophrenic or schizoaffective patients) (151). Larger studies are warranted, although in a study with a substantial sample size the KD reduced schizotypy traits in the general population (166).

Relating to hyperglycemia, diabetes is associated with a higher incidence of several mood and psychiatric disorders (167, 168). A meta-analysis found a significant association between depression and both type I and type II diabetes (169). In diabetic individuals, hyperglycemia is associated with depression (170) and feelings of anger and sadness (171), which may be worse in type I diabetes (170). Such effects are not limited to diabetic patients: hyperglycemia is related to higher readmittance to psychiatric hospitalization (172), and high insulin levels in youth raise the odds of psychosis in young adulthood (173). On more acute timescales, there is some evidence for high glycemic variability relating to low quality of life and negative mood in diabetic patients (174–176), although other studies have not found support for this association (177, 178). Notably, high dietary sugar intake is associated with depression and anxiety in non-diabetic individuals (179–181). These associations do not determine causation but, intriguingly, there are suggestions that depression in the elderly might predispose the development of type II diabetes (182, 183). KDs minimize dietary sugar intake, and provided a stable, mild hypoglycemia which should counteract these deleterious effects on mood. A recent review outlined the heightened risk of dementia in type II diabetes, and the use of KDs as a preventative treatment (184).

Cerebral hypometabolism/hypoperfusion is known to factor into cognitive problems in Alzheimer’s disease, dementia, and mild cognitive impairment; ketogenic strategies can overcome this problem by delivering high energy fuels (ketone bodies) directly to neural tissue (82). A recent report showed that KD or ketone body treatment restores long-term potentiation in a mouse model of Alzheimer’s disease (185). A number of clinical studies have applied the KD (or the modified Atkins diet, also very low carbohydrate) to these disorders (186). Although cognitive tests differ between study groups, the KD is generally found to benefit general cognition, learning and memory, quality of life, general functioning, and mood (187–191). In one study, serum ketones were found to positively correlate with benefits in long term memory (17). Other studies have more directly induced ketosis in these patients with supplements, typically medium-chain triglycerides or ketone esters (which are easily metabolized into ketone bodies), rather than changing diet wholesale. Again, these treatments improved various aspects of cognition (77, 192). A number of studies correlated improved cognition with elevated circulating ketone bodies (193–195) or elevated ketone body uptake in brain (assessed with PET) (196, 197).

There is strong evidence of a metabolic underpinning of ASD, in addition to the genetic and environmental components. For example, this disorder has been found to involve hypoperfusion of specific brain regions (198–200) and to be associated with hyperglycemia, mitochondrial dysfunction, and adenosine dysfunction (201–204). Thus, the KD has multiple mechanisms by which it might be beneficial. KD feeding improved sociability and repetitive behaviors in various animal models of this disorder (26–28, 91, 205, 206). In addition, promising results have been found in autism spectrum disorder patients with KD therapy (207–213).

Addiction and the psychiatric disorders just discussed all have significant co-morbidities; interestingly, KD treatment appears to be helpful with many of these co-morbidities. Diabetes is a co-morbidity in depression, schizophrenia, and Alzheimer’s disease; KD feeding is a greatly beneficial treatment for diabetes (214). Obesity is a co-morbidity in schizophrenia and Alzheimer’s disease; KD feeding is an effective treatment for obesity (215). Attention deficit/hyperactivity disorder is a co-morbidity in addiction and autism spectrum disorder; KD feeding improves attention during treatment of epileptic patients (31, 216–218). Hyperactivity was improved during treatment in epilepsy and autism spectrum disorder (210, 219), but has not been established as a treatment in a non-epileptic clinical ADHD population. Depression is a co-morbidity in addiction and Alzheimer’s disease; KD feeding is effectively antidepressant in non-epileptic populations (11, 117, 144). For rarer co-morbidities such as personality disorder, KD effects remain unknown.

The KD can have some effects on lipids which can be seen as possible negative side effects. However, mild hyperlipidemia was associated with better anticonvulsant effects (219). Even though low-density lipoprotein-C was higher in KD-fed patients, there was no increased coronary plaque burden compared to matched controls (220), and low-density lipoprotein-C levels are generally poorly predictive of cardiovascular disease risk (221, 222).

Taken together, the relationship between metabolic health and a broad range of neurological conditions is emerging, including mental health. The relationship among adenosine, dopamine, and ketogenic metabolic therapy is primary because of the ability to link cell energy, neuronal signaling, and gene expression for both short and long-term effects in key brain areas. The opportunity for metabolic approaches to address multiple comorbidities at once is gaining acceptance. As noted herein there is a wide range of mechanisms and impacts, and reversing and preventing metabolic dysfunction has enormous potential for all ages. However, the opportunity to restore a lifetime of brain health for young people – who may be suffering from mental illness, drug addiction, or both – should be an enormous motivation for continued attention to this field.
